# The Value of Gutta-Percha in Dentistry

**Published:** 1902-06-15

**Authors:** 


### THE VALUE OF GUTTA-PERCHA IN DENTISTRY.

I am decidedly of the opinion that many of the gutta-percha preparations have a real therapeutic value in the treatment of carious and sensitive dentin. This is, I think, markedly true of the base-plate variety, by reason of the vermilion which it contains in addition to the zinc oxid. Although vermilion is assumed to be an insoluble, and hence, therapeutically, an inert substance, it is quite among the probabilities that under the conditions of its incorporation with gutta-percha, and especially under the influence of the heat to which gutta-percha preparations are often subjected in softening them for filling purposes, some decomposition of the mercuric sulphid may occur; with the resultant release of mercury either in the metallic state, or, more probably, as an oxid. Either of these forms would be readily convertible by the oral secretions into a mercury salt having antiseptic qualities. The bluish-black discoloration so generally seen on the surface of base-plate gutta-percha when slightly overheated is unquestionably due to the development of mercury or of an oxid of mercury from the vermilion, the sulphur having been driven off by heat.

In any case it is certain that neither vermilion nor zinc oxid make good culture media for the caries fungus, and to their inhibitory influence their tooth-saving properties must, in some measure at least, be due. The negative electro-chemical relation of gutta-percha is probably also an important factor.

Vermilion also imparts to gutta-percha certain important physical properties, such as increased plasticity when heated, combined with increased toughness at ordinary temperatures. These qualities add greatly to the value of base-plate gutta-percha as a filling material for temporary purposes, and make it especially valuable for use in molar or bicuspid proximal cavities where temporary fillings are desired, with increased space for more permanent operations. The cavities and interspace being thoroughly filled with base-plate gutta-percha, the impact of mastication will usually in a few weeks effect painlessly the separation desired. This was a detail of practice on which the late Dr. Bonwill laid much stress and from which he secured excellent results, especially in the preparation of cavities for filling with plus contours.—Dr. Litch, in *Dental Brief*.

